# Juvenile Male Rats Exposed to a Low-Dose Mixture of Twenty-Seven Environmental Chemicals Display Adverse Health Effects

**DOI:** 10.1371/journal.pone.0162027

**Published:** 2016-09-06

**Authors:** Niels Hadrup, Terje Svingen, Karen Mandrup, Kasper Skov, Mikael Pedersen, Hanne Frederiksen, Henrik Lauritz Frandsen, Anne Marie Vinggaard

**Affiliations:** 1 Division of Diet, Disease Prevention and Toxicology, National Food Institute, Technical University of Denmark, Søborg DK-2400, Denmark; 2 Division of Food Chemistry, National Food Institute, Technical University of Denmark, Søborg DK-2400, Denmark; 3 Department of Growth and Reproduction, Copenhagen University Hospital (Rigshospitalet), Copenhagen DK-2100, Denmark; University of Missouri Columbia, UNITED STATES

## Abstract

Humans are exposed to a large number of environmental chemicals in their daily life, many of which are readily detectable in blood or urine. It remains uncertain if these chemicals can cause adverse health effects when present together at low doses. In this study we have tested whether a mixture of 27 chemicals administered orally to juvenile male rats for three months could leave a pathophysiological footprint. The mixture contained metals, perfluorinated compounds, PCB, dioxins, pesticides, heterocyclic amines, phthalate, PAHs and others, with a combined dose of 0.16 (Low dose), 0.47 (Mid dose) or 1.6 (High dose) mg/kg bw/day. The lowest dose was designed with the aim of obtaining plasma or urine concentrations in rats at levels approaching those observed in humans. Some single congeners were administered at doses representative of combined doses for chemical groups. With this baseline, we found effects on weight, histology and gene expression in the liver, as well as changes to the blood plasma metabolome in all exposure groups, including low-dose. Additional adverse effects were observed in the higher dosed groups, including enlarged kidneys and alterations to the metabolome. No significant effects on reproductive parameters were observed.

## Introduction

Humans are continuously exposed to a large number of chemicals from various sources, including consumables, cosmetics, fabrics and polluted air. Most of these chemicals are typically present in our bodies at very low concentrations and usually deemed safe. ‘Safe levels’ of chemical exposure, however, are normally based on the toxicological profile of one chemical at a time. What happens when a substantial number of chemicals are present simultaneously, but still at very low levels, remains less understood and could potentially have unforeseen consequences for humans and the environment.

*In vivo* studies designed to determine potential effects of large chemical mixtures, particularly at low doses, are few. Hence, more *in vivo* mixture studies would greatly aid in our understanding of complex chemical interactions in intact biological systems. But we can gain some early insights from studies already performed. For instance, we previously showed that perfluorononanoic acid (PFNA) at a high-end human exposure range can exert mixture effects in rats when given in combination with a 14-chemical mixture [1]. Further examples include: increased lytic activity by natural killer cells following low level exposure to 18 persistent contaminants [2]; increased prostate inflammation in rats with an atrazine metabolite mixture at a dose of 0.09 mg/kg bw/day [3]; changes to blood cell counts in mice following exposure to a mixture of six pesticides at doses derived from the acceptable daily intake levels in humans [4]; decreased red blood cell count and hemoglobin concentrations in mice after exposure to a mixture consisting of 4, 5 and 8 μg/kg bw/day of atrazine, endosulfan and chlorpyrifos, respectively [5], and; additive effects of thyroid disrupters in rats exposed at a dose corresponding to the environmental background [6]. These studies not only show low-dose mixture effects of chemicals when present together, but also highlights the importance of taking putative mixture effects into account when extrapolating safe levels of exposure to bioavailable chemicals.

Mixture studies have typically involved simultaneous exposure to closely related chemicals, for instance pesticides, thyroid disruptors or atrazine metabolites. In a human context, however, the chemical burden is far more complex and includes chemicals from vastly different sources and classifications. Therefore, we designed a low-dose mixture study in juvenile male rats using 27 chemicals relevant for human exposure. We chose to expose juvenile rats since this life stage represents a sensitive period of male reproduction. Although the male offspring are born with fully differentiated testes comprising the main cellular constituents [7], some key events take place in postnatal life leading up to puberty. Firstly, the fetal Leydig cell population is replaced by adult Leydig cells [8], and secondly, the germ cells exit mitotic arrest and develop within the seminiferous tubules to the point of spermatogenesis during puberty [9]. Thus, although most studies aimed at examining endocrine disrupting effects of chemicals on late-life male reproductive health, the juvenile stage is a sensitive period that warrants further scrutiny.

Data from the National Health and Nutrition Examination Survey study [10] and other sources were used to identify the chemicals and their corresponding exposure levels in the human population. We chose to combine 27 chemicals and expose the animals for 3-month to represent chronic exposure. An additional two doses of 3- and 10-fold higher were also included. The lowest dose was designed with the aim of obtaining rat plasma or urine concentrations approaching those reported in humans. Single congeners were in some cases selected to cover chemical groups. Furthermore, doses were determined using linear extrapolation of doses from high-dose animal studies. In other words, some assumptions where made that can give rise to some uncertainty when extrapolating data for human relevance. Nevertheless, we cautiously designate the mixture “a low-dose mixture”. After three months of exposure the animals were assessed for general pathology, metabolic changes, as well as more thorough histological and molecular analyses of affected organs.

## Materials and Methods

### Chemicals

A mixture comprising 27 chemicals was designed based on information on human exposure levels according to the NHANES database (geometric means) [10] and other sources (Table 1). Some chemicals were chosen to represent certain groups of chemicals: Acrylamide, to also represent the metabolite glycidamide; Bisphenol A to represent bisphenols; Perfluorooctanesulfonic acid (PFOS) and PFNA to represent perfluorinated compounds; Mono-n-butyl phthalate to represent mono- and di-ester phthalates; PCB-153 to represent PCBs; TCDD to represent dioxins; Benzo[a]pyrene to represent PAHs; PHIP and MeIQx to represent heterocyclic amines. For each chemical, rat or human bioavailability studies were used to estimate the doses needed for rats to reach a plasma or urine concentration similar to those reported in humans according to the NHANES database and others. We assume that urinary or plasma levels are linearly related to dose levels For mono-n-butyl phthalate, AHTN, PHIP and MeIQx, human intake levels were used to assess doses to the rats and multiplied with a factor of 2.2 to account for the difference in metabolic rate between humans and rats (body surface area conversion) [11]. The chemicals are presented in Table 1 and the calculations underlying the doses in Supporting information (S1 Table). Chemicals were purchased from Sigma Aldrich except; benzophenone-3 (AcrosGeel), Bisphenol A and DDE (LGC Standards), Triclosan (AlfaAesar.), TCDD (AccuStandard), Benzo[a]pyrene (Lancaster), MeIQx and PHIP (Abcam).

**Table 1:** Chemicals included in the mixture.

Chemical (common name / /chemical name)	CAS number	Concentration measured in humans (reference)	Dose in Low-dose animal group (μg/kg bw/day)
Acrylamide	79-06-1	5.4 nmol/L plasma [10]	4
Benzophenone-3 /oxybenzone	131-57-7	22.9 μg/L urine [10]	2.6
Bisphenol A	80-05-7	2.64 μg/L urine [10]. Alternatively a plasma concentration in the Hong Kong population was 0.95 μg/L [12]	10
Triclosan	3380-34-5	13 μg/L urine [10]	5
Ortho-phenylphenol	90-43-7	0.5 μg/L urine [10]	0.06
trans-nonachlor	39765-80-5	0.11 ng/g serum [10]	0.35
p.p-DDE / Dichlorodiphenyldichloroethylene	72-55-9	1.54 ng/g serum [10]	13
2.4.6-trichlorophenol	88-06-2	2.85 μg/L urine [10]	10
Chlorpyrifos	2921-88-2	1.77 μg/L urine of the metabolite TCPγ. [10]	0.4
3-phenoxybenzoic acid	3739-38-6	0.29 μg/L urine [10]	0.01
Arsenic	1327-53-3	8.3 μg/L urine [10]	1.2
Barium	10361-37-2	1.5 μg/L urine [10]	0.13
Cadmium	10108-64-2	0.41 μg/L blood [10]	0.1
Cesium	7647-17-8	4.4 μg/L urine [10]	0.48
Cobalt	7646-79-9	0.38 μg/L urine [10]	0.05
Lead	7758-95-4	0.80 μg/L urine [10]	16
Mercury	7487-94-7	0.44 μg/L urine [10]	3.5
Thallium	7791-12-0	0.18 μg/L urine [10]	0.3
PFOS / Perfluoro-octanesulfonic acid	111873-33-7	20.7 μg/L serum [10]	0.9
PFNA / Perfluoro-nonanoic acid	375-95-1	1.0 μg/L serum [10]	0.2
Mono-n-butyl phthalate	131-70-4	24.6 μg/L urine [10]	62
AHTN / 6-Acetyl-1,1,2,4,4,7-hexamethyltetraline	1506-02-1	No data on human tissue concentrations. Values are based on intake HERA (2004) data [13]	6.2
PCB 153 (covering the PCBs) /Polychlorinated biphenyl 153	35065-27-1	0.17 ng/g serum [10]An alternative number is reported by Bakker *et al*. [14]. Here seven indicator PCBs are present at 0.36 ng/g serum	20
TCDD (Dioxines) / 2,3,7,8-Tetrachlorodibenzo-p-dioxin	1746-01-6	2.6 ng/L serum (sum of four dioxins: HpCCD 155 fg/g serum; HxCCD 105 fg/g serum; OCDD 2230 fg/g serum; HpCDF 62 fg/g serum) [10]	0.034
Benzo[a]pyrene (PAHs)	50-32-8	6.3 μg/L urine(a sum of 10 PAHs: 2-hydroxyfluorene 304 ng/L; 3-hydroxyfluorene 134 ng/L; 9-hydroxyfluorene 267 ng/L; 1-hydroxynaphtalene 2680 ng/L; 2- hydroxynaphtalene 2470 ng/L; 1-hydroxyphenantrene 140 ng/L; 2-hydroxyphenantrene 54 ng/L; 3-hydroxyphenantrene 105 ng/L; 4-hydroxyphenantrene 23 ng/L; 1-hydroxypyrene 89 ng/L) [10]	0.4
PHIP / 2-Amino-1-methyl-6-phenylimidazo(4,5-b)pyridine	105650-23-5	0.41 ng/ L (mean of 13 subjects, 24 h urine set to 1 L) based on Wakabayashi *et al*. [15] [15][15]	0.1
MeIQx /2-Amino-3,8-dimethylimidazo[4,5-f]quinoxaline	77500-04-0	22.5 ng/ L (mean of 13 subjects, 24 h urine set to 1 L) based on Wakabayashi *et al*. [15]	0.05
**Total dose**			**160**

### Animal procedure

Animal experiments were carried out in accordance with the guidelines stipulated by the National Food Institute’s Animal Welfare Committee. Ethical approval was given by the Danish Animal Experiments Inspectorate (Authorization no. 2012/561-188). The animals were monitored twice daily for the duration of the exposure experiment. Clinical assessments included general well-being, feed and water intake, as well as observation of presence and appearance of feces. If an animal was to appear clinically sick based on the monitoring criteria, considered highly likely to die within 24 h, or otherwise deemed unethical to keep alive, it was to be humanely euthanized immediately. No unexpected deaths occurred during the experiments, but animals from the High-dose group were prematurely euthanized as some animals started to display poor clinical appearance before the end of the dosing period.

Four week-old male Wistar Hannover Galas rats were purchased from Taconic European and allowed to acclimatize for two weeks. Animals were housed in pairs in Makrolon type III cages (Bioscape) with Tapvei bedding (Tapvei) and *ad libitum* access to Altromin 1324 feed (Altromin) and acidified tap water in bisphenol A-free bottles. Lights were on from 9 am to 9 pm. The temperature was 22±1°C with a humidity of 55±5%. The rats were randomly assigned to the following groups (n = 10 for each group): i) control group receiving vehicle, ii) Low-dose mixture receiving in total 0.16 mg/kg bw/day, iii) Mid-dose mixture given in total 0.47 mg/kg bw/day and iv) High-dose mixture given in total 1.6 mg/kg bw/day. The chemicals were administered orally by gavage once daily using corn oil as vehicle for 14 weeks. The dose volume was 2 mL/kg bw. Body weight, feed, water intake and overall clinical appearance were recorded during the experiment. Blood samples (~0.2 mL) were drawn from the lingual vein in randomized order at day 30 and 66 (denoted day 60 in figures) for all animals. At the end of the experimental period, animals were dosed 1½ h before being anaesthetized in CO_2_/O_2_ and decapitated in a randomized order. Trunk blood was collected, kidney, liver, testicles, retroperitoneal fat pad, prostate, heart, adrenals, brain, pituitary and spleen were dissected and weighed, before being processed for histology or other downstream experiments. Plasma was isolated from the blood samples by centrifugation at 1,700 g for 10 min at 4°C and stored at -80°C until metabolomics and internal dose analyses.

A separate single-dose experiment was conducted to measure the relative excretion of selected chemicals in urine samples collected over 24 h after excretion. Four week old male Wistar Hannover Galas rats were housed in metabolic cages after having received a single dose of vehicle (corn oil; control), Low- or Mid-dose as stipulated above. Each group consisted of 2 animals. The urine was continuously collected into vials on ice. Two additional ‘0’-control samples (~field blanks) consisting of 5 mL synthetic urine was collected to measure background levels of chemicals. This was done by passing 2.5 mL synthetic urine through a metabolic cage at 4 hours and repeated at 22 hours, then collected and processed as for the biological samples. The urine was stored at -80°C until further analysis.

Animal body and organ weights were tested for normal distribution using the D’Agostino & Pearson omnibus normality test using Graph Pad Prism V5 (Graph Pad, La Jolla, CA, USA). Data were tested by ANOVA, or in case of no normal distribution, by the Kruskal-Wallis test. Dunnett’s (ANOVA) or Dunn’s (Kruskal-Wallis) post tests were applied to determine the effects of single treatment groups compared to controls.

### Urinary excretion measurement

The uptake and subsequent excretion of benzophenone-3, triclosan, and mono-n-butyl phthalate in 24 h urine samples were deconjugated by enzymatic hydrolysis and measured by isotope diluted TurboFlow-LC-MS/MS as previously described [16,17].

### Histology

Formaldehyde-fixed livers and kidneys were sectioned at 3 μm thickness and stained with hematoxylin and eosin. Evaluation of sections was performed blinded to treatment groups. Livers were assessed for signs of hepatocellular fatty changes (macrovesicular changes with displacement of the nucleus, microvesicular changes), localization of the changes (centrilobular, midzonal, periportal or intermediate localizations) and severity of the vacuolar changes defined as the distribution of changes in hepatocytes (scored from minimal to severe). Kidneys were evaluated for hypertrophy of convoluted tubules. Scores for kidneys were i) no hypertrophy, ii) slight hypertrophy, and iii) hypertrophic convoluted tubules.

Statistical analyses of histology scoring data were performed with SAS Enterprise Guide 6.1 using a Fisher’s exact test. When more than 2 score-levels were used, an overall Fisher’s exact RxK test was used to assess the distribution of scores. Additionally, a Fisher’s 2xk test (including all score-levels) was run to identify differences from controls in each exposure group. A 2x2 Fisher’s exact test was performed for dichotomous data (i.e. two score-levels, e.g. for presence of microvesicular changes and presence of macrovesicular changes). As expected, no changes were observed in controls, and a one-sided 2x2 Fisher’s exact test was performed for the presence of macrovesicular or microvesicular changes in hepatocytes.

### Hormone measurements

Blood samples were analyzed for steroid hormone levels by LC-MS/MS as previously described^1^.

### Rat hepatotoxicity gene array

The Rat Hepatotoxicity RT2 Profiler^™^ PCR Array (Qiagen; PARN-093ZE-4) was used to profile expression of 84 genes related to hepatotoxicity. Tissue samples derived from 4 randomly chosen livers each from control, Low- and Mid-dose groups. The High-dose group was not included as it was prematurely terminated, making direct comparisons unfeasible. Total RNA was extracted from homogenized rat livers using the RNeasy Mini kit (Qiagen) including on-column DNase I treatment. cDNA was synthesized from 1 μg total RNA using the recommended RT^2^ First Strand Kit (Qiagen). Samples were prepared for 384-well format and run on a 7900HT Fast Real-Time PCR System (Applied Biosystems). Relative transcript levels were determined by the comparative Ct-method using the Qiagen on-line Data Analysis Center. Data was normalized with the geometric mean of four reference genes: *Actb*, *Hprt1*, *Ldha* and *Rplp1*.

### RT-qPCR TaqMan analysis

The protocols were essentially as described previously [18], with 500 ng total RNA used to synthesize cDNA. Samples derived from 4 randomly selected livers from each study group (control, Low- and Mid-dose) and were different from those used for the gene array analysis. cDNA was diluted 1:20 and used at 3 μl per RT-qPCR reactions. TaqMan Gene Expression Assays (Life Technologies) were: *Abcc3* (Rn01452854), *Actb* (Rn00667869), *Aldo1* (Rn00820577), *Car3* (Rn01461970), *Cdkn1a* (Rn01427989), *Cyp1a2* (Rn00561082), *Fmo1* (Rn00562945), *Nqo1* (Rn00566528), and *Rps18* (Rn01428913). RT-qPCR assays were run on a 7900HT Fast Real-Time PCR System (Applied Biosystems). Relative transcript abundance was calculated by the comparative Ct-method using the reference genes *Rps18* [18] and *Actb* (Qiagen). Statistical comparisons were performed using unpaired, two-tailed Student’s t-test.

### Metabolomics

The blood plasma analysis was conducted as previously described [19]. Phospholipids, the non- polar fraction as well as the polar fraction were recovered. Metabolites were separated using an Ascentis Express C8 HPLC column (Supelco) and measured using a Bruker maXis time-of-flight mass spectrometer equipped with an electrospray ion source (Bruker Daltonics). The samples were analyzed in both positive and negative ionization mode, and data obtained as mass-to-charge (m/z) ratios with concomitant HPLC metabolite retention times. The Profile Analysis 2.1 software package (Bruker Daltonics) was used for data analyses. Data buckets had a time window of 60–720 s and an m/z ratio of 100–700 using the ‘find molecular feature’-algorithm, including time alignment. Data noise was reduced using the software program R [20]. The raw intensity data were transferred to the online MetaboAnalyst server 7 [21], normalized by sum and log-transformed. Pareto-scaling and Partial least squares (PLS) discriminant analysis were performed collectively for all data sets. Discriminatory metabolites were determined by use of a false discovery rate-adjusted p-value of 0.05 following ANOVA analysis. Raw non-transformed or log-transformed data of these metabolites were tested for normal distribution using the D’Agostino & Pearson omnibus normality test. Data were then tested by ANOVA or, if not normally distribution, by the Kruskal-Wallis test. Dunnett’s (ANOVA) or Dunn’s (Kruskal-Wallis) post tests were applied to determine the effects of single treatment groups. Attempts to identity single metabolites was done by use of the Human Metabolome Database 10 [22] searching the accurate mass of the metabolites. In addition, adducts or fragments (e.g. plus Na^+^ or minus H_2_O) at identical HPLC retention times were considered.

## Results

### Body weight, clinical appearance, lethality and organ weights

**Fig 1:**
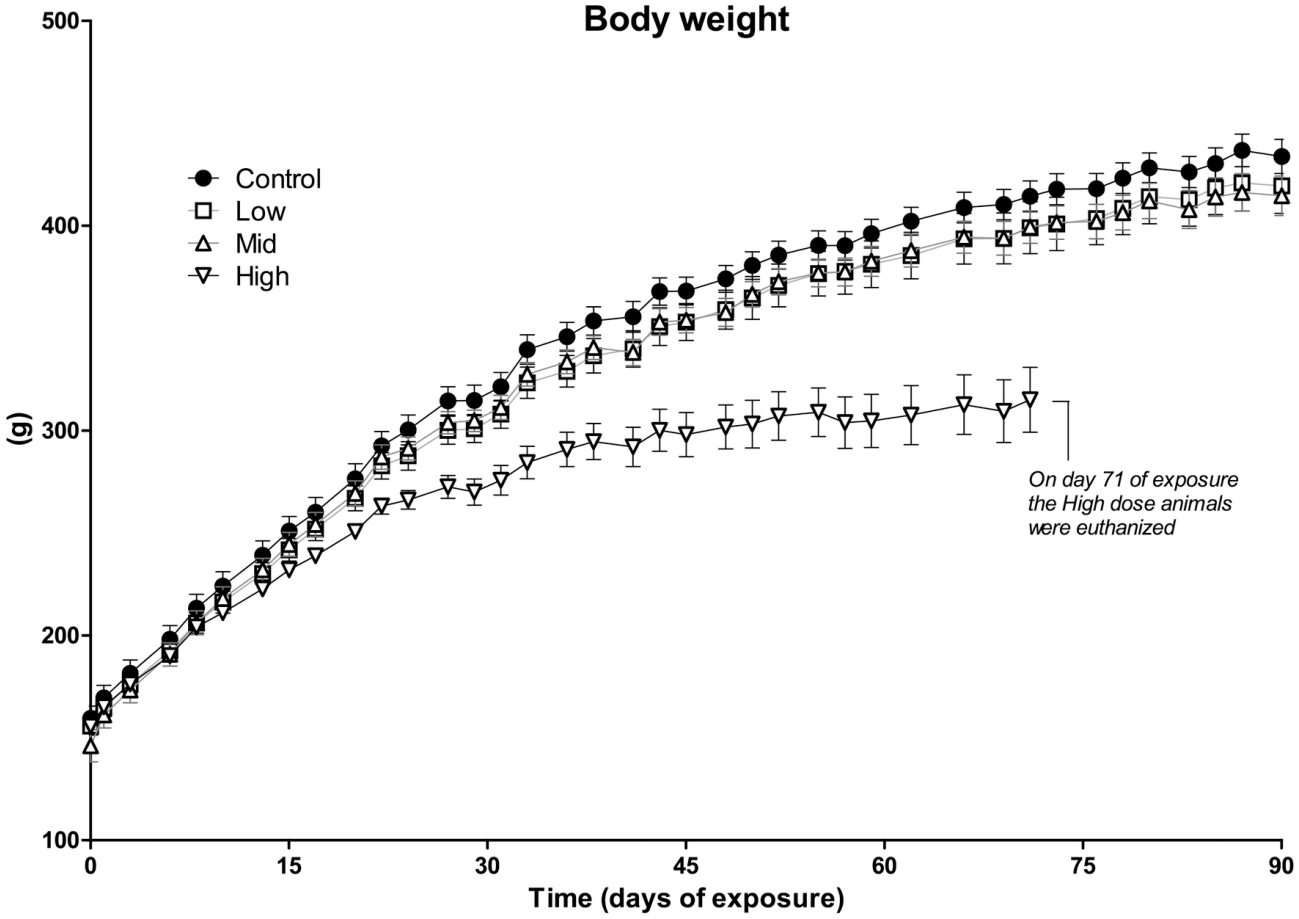
Absolute body weight of rats following exposure to a 27-chemical mixture. Juvenile rats were given an oral daily dose of a 27 chemical mixture for 3 months. Animals in the High-dose group were euthanized prematurely (day 71) due to poor clinical appearance. No significant differences in body weight were observed in other dose groups compared to control animals. Doses were: Low = 0.16 mg/kg/day; Mid = 0.47 mg/kg/day; High = 1.6 mg/kg/day. N = 10 for each group. Data are presented as mean ± SEM.

**Fig 2:**
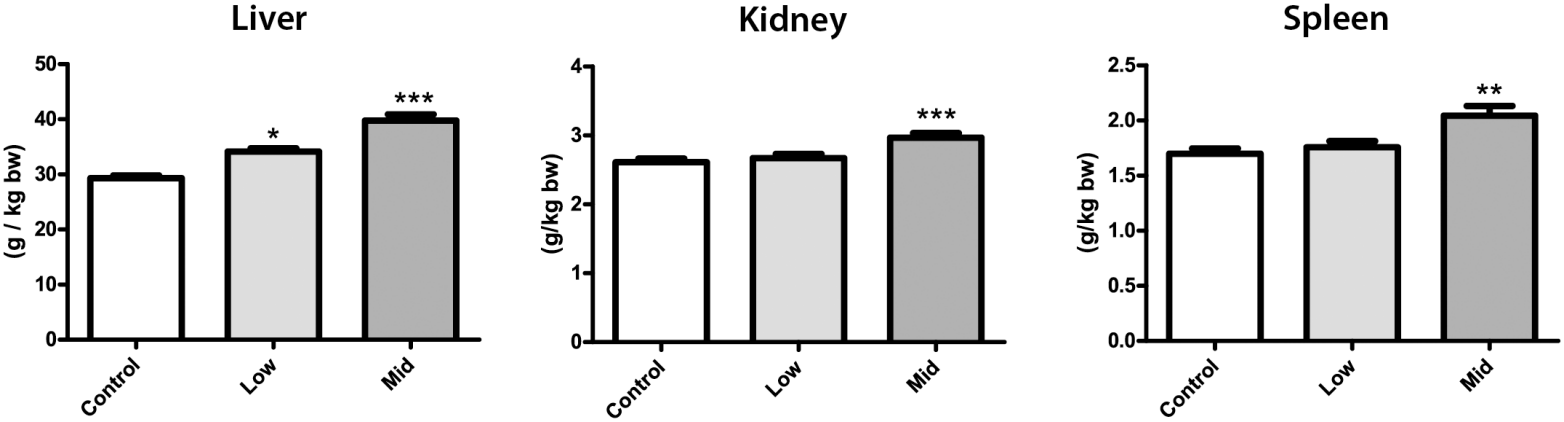
Relative organ weights of animals. Relative weights of liver, kidney and spleen in rats administered Low- and Mid-dose of the 27 chemical mixture. In the Mid-dose group, all three organs showed significantly increased weight, whereas only the liver was significantly larger in the Low-dose group. N = 10 for each group, Data are presented as Mean ± SEM. Statistical tests were one-way ANOVA with Dunnett’s post-test for normal distributed data or with Kruskal-Wallis with Dunn’s post-test for data that were not normal distributed (*p <0.05, **p<0.01 and ***p<0.001).

The average body weight of animals in the High-dose group was approximately 20% lower than that of the control group on exposure day 71 (Fig 1). As this could indicate severe toxicity, the animals from the High-dose group were euthanized on day 71, 19 days prematurely (One animal from the High-dose group was euthanized already on day 60 due to a poor clinical appearance). There was also a tendency towards reduced body weight in the Low- and Mid-dose groups compared to controls, albeit not statistically significant at the end of the exposure period (Fig 2). Feed and water intake was lower for the High-dose group, but not different for the Low- and Mid-dose groups relative to controls (data not shown). At the organ level, we observed a significant increase in relative liver weight in both the Low- and Mid-dose groups (Fig 2). Relative kidney and spleen weights were significantly increased in the Mid-dose, but not Low-dose group (Fig 2). Organ weights were not measured for the High-dose group, as these animals were euthanized prematurely.

### Single dose experiment

Triclosan, benzophenone-3 and mono-n-butyl phthalate were measured in 24 hour urine samples collected after a single administration of no-dose (controls), Low- and Mid-dose. The concentrations of triclosan (Low-dose: 0.30 and 0.49 ng/mL; Mid-dose 7.03, 5.98 ng/mL), benzophenone-3 (Low-dose: 5.0 and 11.2 ng/mL; Mid-dose 14.7 and 30.4 ng/mL) and mono-n-butyl phthalate (Low-dose: 314 and 617 ng/mL; Mid-dose 816 and 1625 ng/mL) varied between the two dosed rats in each dose group. However, after adjustment for individual rat weight, volume of urinary excretion and osmolality, the urinary recoveries of benzophenone-3 and mono-n-butyl phthalate were calculated to be 7–10% and 19–23%, respectively in both the Low- and Mid-dosed rats. Only 0.2% (Low-dose) and 1–2% (Mid-dose) of triclosan was excreted in urine. In control rats the concentrations in 24 hour urine samples were below LOD for triclosan, 0.22 ng/mL for benzophenone-3, whereas a weak mono-n-butyl phthalate contamination of 16.1 and 14.2 ng/mL was observed.

### Histology

**Fig 3:**
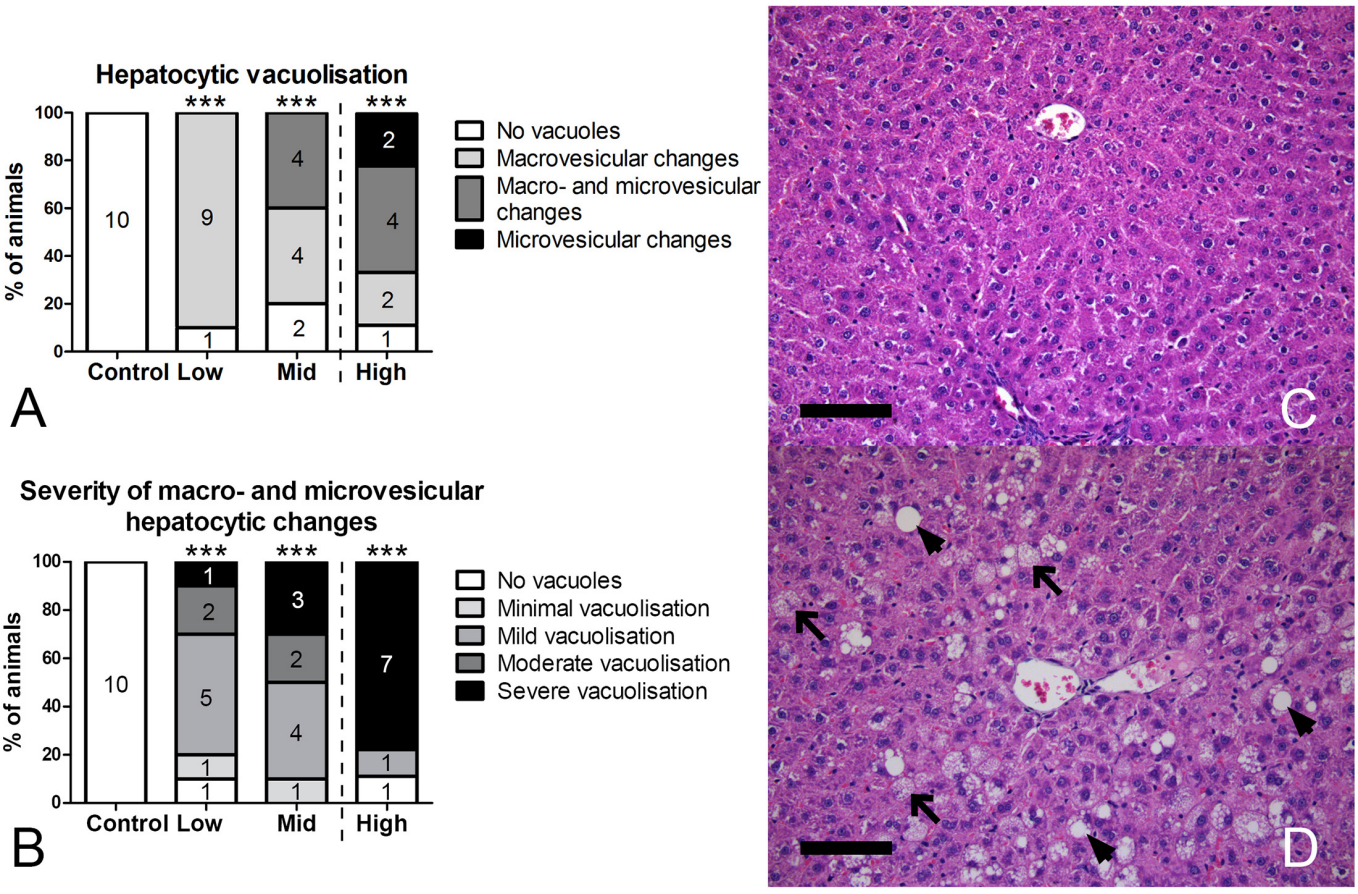
Type (A) and severity (B) of histological changes of the liver. A, Significant hepatocellular vacuolization was observed in livers in all dose groups, with only macrovesicular changes observed in Low-dose, but microvesicular changes appearing already in Mid-dose and occurring more frequently in High-dose. B, Severity of hepatocellular vacuolization, including both macrovesicular and microvesicular changes, increased with increasing dose. C, Liver from control rat. D, Liver from Mid-dose group showing moderate vacuolization with macrovesicular (arrowheads) and microvesicular (arrows) changes. N = 9–10/group. ***p<0.001 in Fisher’s exact 2xk test. Scale bar = 100 μm.

Since several livers showed enhanced lobular pattern at the macroscopic level, livers were evaluated histologically (Fig 3). Hepatocytic accumulations were observed in exposed animals. Macro-vesicular changes in hepatocytes were evident in 9 out of 10 animals already at Low-dose exposure. The rate of hepatocellular vacuolization was comparable in Mid- and High-dose groups, but now also showing clear signs of both macro- and micro-vesicular changes (Fig 3A and 3D). Micro-vesicular changes are suggestive of more severe hepatotoxicity and were observed in the Mid- and High-dose groups only, with a greater incidence rate in High-dose (40% of animals with p = 0.04 for the Mid-dose and 67% of animals with p = 0.003 for the High-dose in a one-sided 2x2 Fisher’s exact test; Fig 3A). Livers with micro-vesicular changes in the High-dose group often displayed ballooning of hepatocytes in periportal areas or changes occupying most of the lobules (diffuse). The severity of vacuolization increased with increasing dose, with more hepatocytes being affected (Fig 3B). This was evident by a shift from minimal or mild in 67% and 50% of animals with vacuolated hepatocytes (macro- and/or micro-vesicular) in Low- and Mid-dose groups respectively, to 88% of affected animals in the High-dose group with moderate to severe vacuolization (Fig 3B). Hepatic changes were predominantly localized to the mid-zonal area of the lobules.

Kidney histology from the same animals was evaluated, but revealed no significant changes in the Low-dose group relative to control animals (data not shown). In the Mid-dose group, an increase in the incidence of slight hypertrophy of convoluted tubules in the cortex (6 out of 10 animals) was observed (p = 0.057 in a 2-sided 2x2 Fisher’s exact test). In a subset of animals from the prematurely euthanized High-dose group, 5 out of 6 kidneys displayed severe hypertrophy of convoluted tubules (data not shown, p = 0.0002 in a 2-sided 2xk Fisher’s exact test).

### Steroid hormone levels

**Fig 4:**
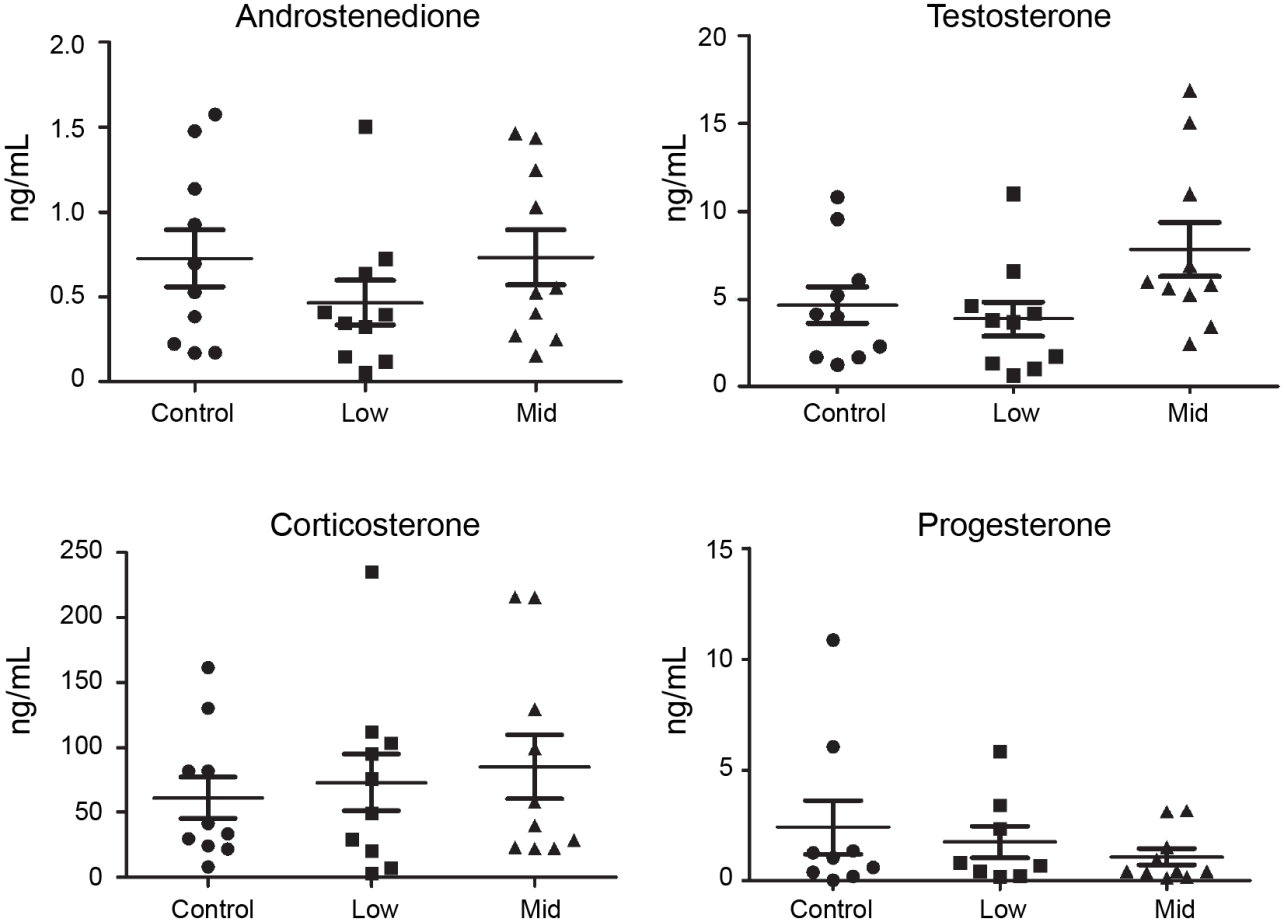
Steroid hormone levels. Steroid hormone levels in serum of rats administered a 27-chemical mixture for 90 days. The levels of androstenedione, testosterone, corticosterone or progesterone were quantified by LC-MS/MS. No statistically significant changes in hormone levels were observed, but testosterone showed a strong trend towards elevated levels in the Mid-dose group relative to controls. N = 10 for each group. Data are presented as mean ± SEM.

Steroid hormone levels were analyzed in blood serum collected after 90 days of chemical exposure. Four hormones were measured; androstenedione, testosterone, corticosterone and progesterone. Although we observed a trend towards elevated testosterone levels in Mid-dose animals (Fig 4), we observed no statistically significant changes in any of the four hormones in Low- or Mid-dose animals compared to controls.

### Hepatotoxicity gene expression array

**Fig 5:**
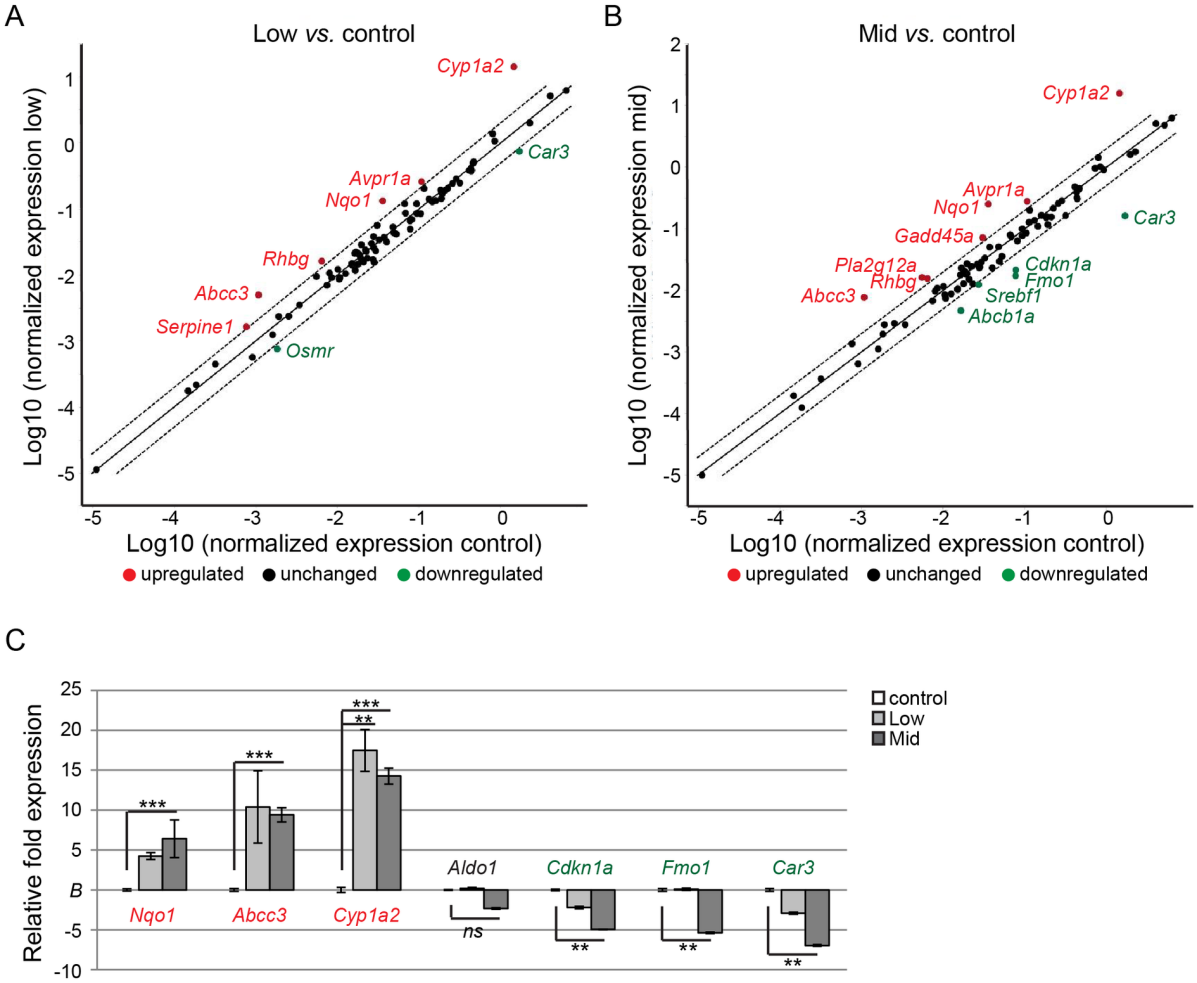
Relative mRNA levels of hepatotoxicity marker genes. **A,B)** Scatter-plots of differentially expressed hepatotoxicity biomarker genes in livers of control animals and animals from Low- and Mid-dose mixture groups. Relative fold expression was determined from average transcript abundance across groups (n = 4 from each group). Genes up-regulated more than 2-fold are highlighted in red and genes down-regulated more than 2-fold highlighted in green. Dashed lines indicate two-fold change in expression relative to controls (whole line). C) The targeted gene array was verified by TaqMan RT-qPCR assays on 7 genes across the animal groups (n = 4 from each group). The identified up-regulated genes *Nqo1*, *Abcc3* and *Cyp1a2*, unaltered gene *Aldo1*, and down-regulated genes *Cdkn1a*, *Fmo1* and *Car3* all showed similar expression profile as from the array. Statistical significance was determined by two-tailed, unpaired Student’s t-test (*p <0.05, **p<0.01 and ***p<0.001).

Next we performed a hepatotoxicity 84-gene expression array on four liver samples from each group (control, Low- and Mid-dose). As depicted in Fig 5A, animals from the Low-dose group displayed altered gene expression at a conservative cut-off value of 2-fold change in transcript abundance. Particularly, a down-regulation of *Car3* and up-regulation of *Gadd45a* and *Nqo1* is indicative of hepatotoxicity, whereas dysregulation of *Abcc3* can indicate cholestasis. A similar profile was observed in the Mid-dose group, but with a slightly higher fold expression relative to control, and with more genes displaying statistically significant changes in relative transcript abundance (Fig 5B). For instance, two additional markers for hepatotoxicity, *Pla2g12a* and *Fmo1* were up- and down-regulated, respectively. There was also a significant down-regulation of *Abcb1a*, an additional marker for cholestasis, as well as *Srebf1*, indicative of steatosis. Also, the necrosis marker *Cdkn1a* was significantly down-regulated in the Mid-dose group. Finally, *Cyp1a1* showed the highest degree of up-regulation, both in Low- and Mid-dose groups (Fig 5A and 5B).

To verify the gene array data, we performed RT-qPCR experiments on additional liver samples obtained from the three groups; control, Low- and Mid-dose (Fig 5C). We selected three genes each that displayed up- or down-regulation in the array, namely *Nqo1*, *Abcc3*, *Cyp1a2* and *Cdkn1a*, *Fmo1*, *Car3*, as well *Aldo1* which did not show a significant change in transcript abundance. All seven genes were verified to display a similar expression profile between the biological samples as was shown with the targeted gene array.

### Metabolomics

**Fig 6:**
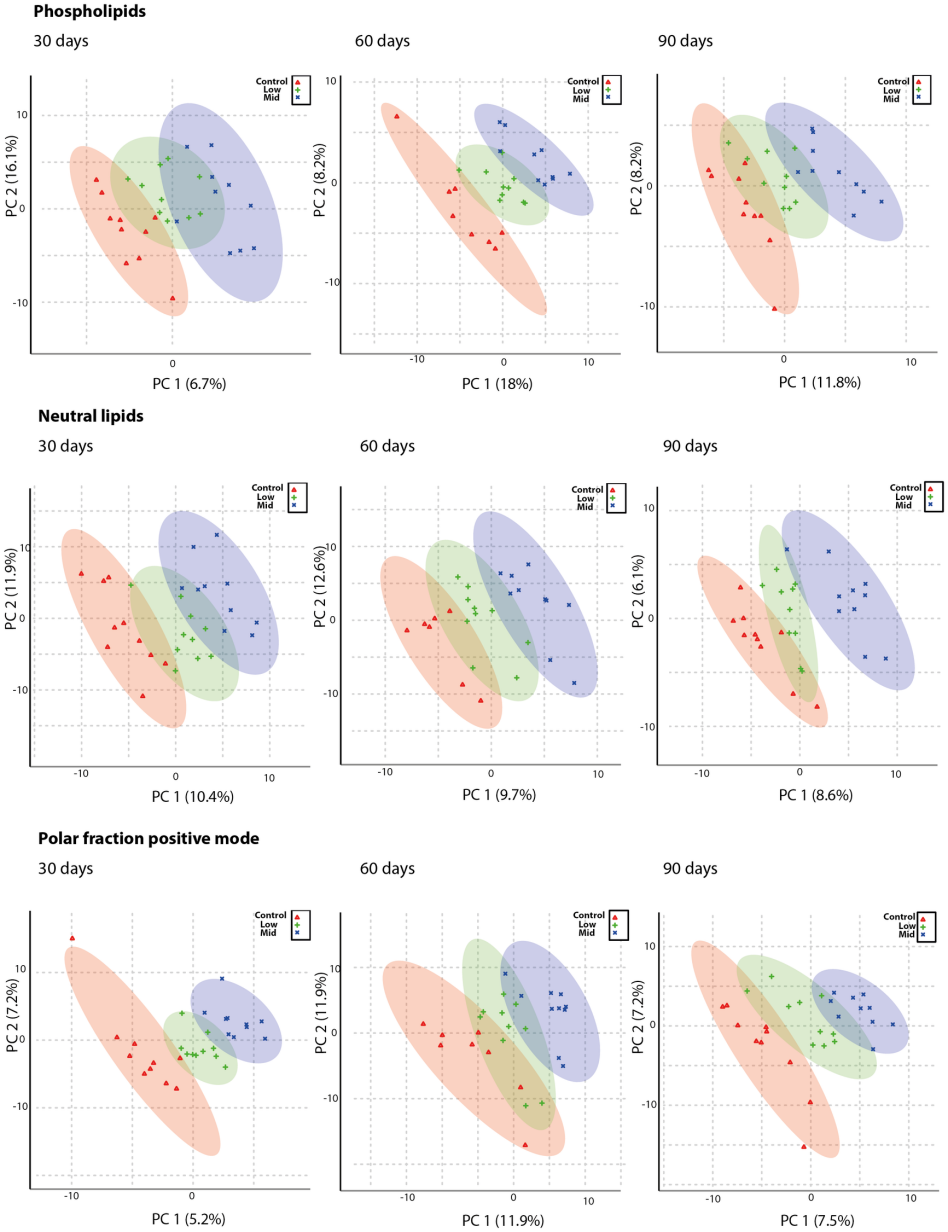
PLS DA plots of metabolomics data show that the Low-dose has an overall effect on the metabolome. Plasma from rats administered Low- and Mid-dose for 30, 60 or 90 days was separated into a phospholipid, a neutral lipid and a polar fraction. These were separated by HPLC and analyzed by MS using the positive ionization mode (positive mode) and for the polar fraction also the negative mode. A clear separation of experimental groups is evident shown as a dose-dependent effect on the metabolome of the mixture and as well as a low dose effect on the metabolome. Red groups are control, green are Low-dose and blue are Mid-dose. Numbers given in brackets at the axes designate the percentages of variation accounted for by the specific principal components (PCs).

Blood samples were obtained throughout the experiment to allow for a temporal metabolomics analysis at 30, 60 and 90 days after initiation of exposure. As animals from the High-dose group were euthanized at 70 days, analyses from this group were only performed at 30 and 60 days and also excluded from the PLS-DA scores plots (Fig 6). Otherwise, a clear separation of control, Low-and Mid-dose groups were observed in the positive mode for phospholipids, neutral lipids and for the polar fraction. In all fractions, the control group was located to the left as compared to the Low- and Mid-dose groups in the scores plots. For the polar fractions analyzed in negative mode, the separation was less clear (data not shown). To identify metabolites that were differently regulated in response to chemical exposure we used a false discovery rate corrected ANOVA in the Metaboanalyst web-tool with subsequent evaluation of raw data in Graph Pad Prism. By searching the HMDB database for exact mass of metabolites from the lipid and polar fractions alongside searches for fragments and adducts, putative identities of metabolites were obtained (S2 Table).

**Fig 7:**
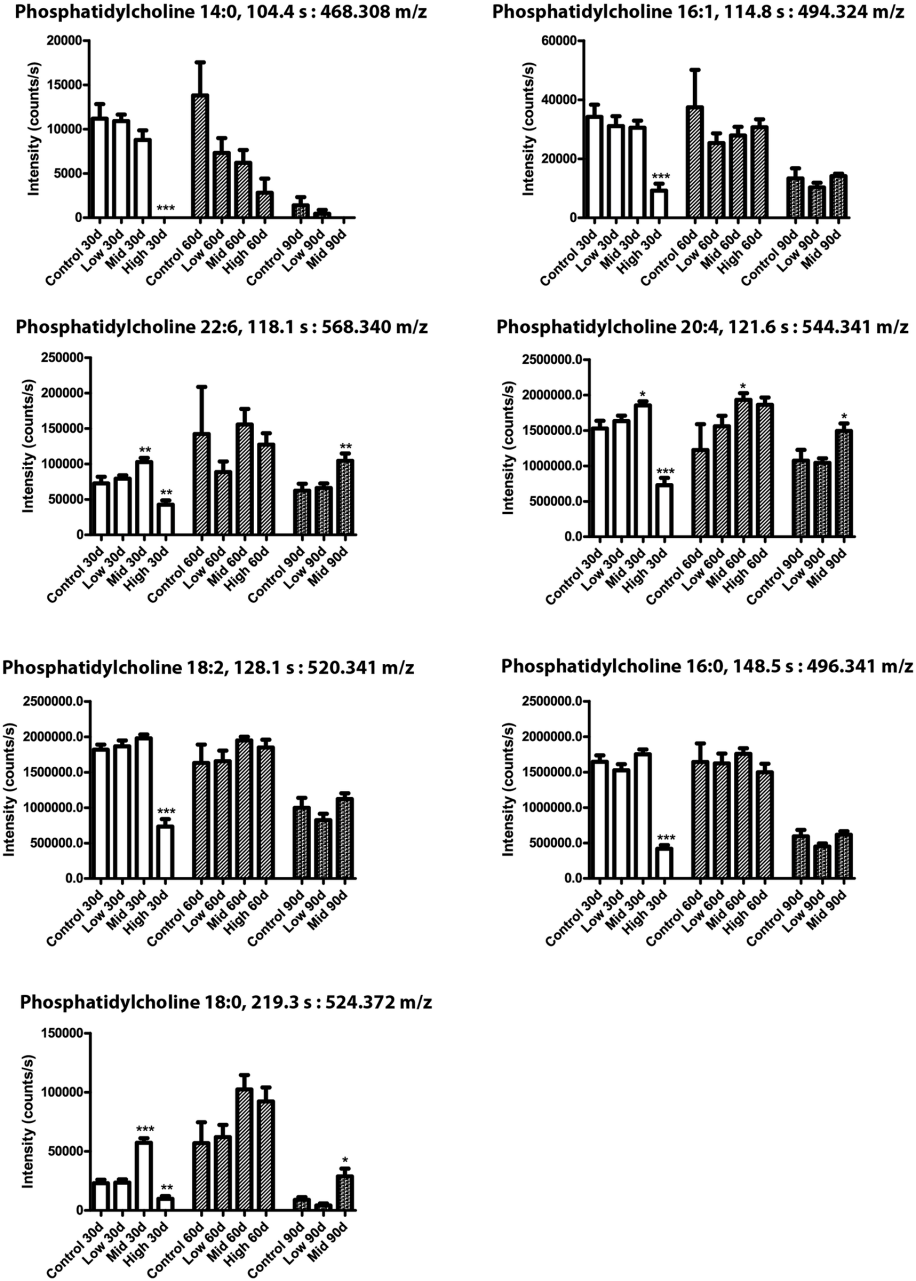
Phospholipid levels were altered by the mixture. Metabolome analysis of rat blood after 30, 60 or 90 days of exposure to a 27-chemical mixture. N = 10 for each group and data are means ± SEM. The data were statistically tested as the exposure groups versus controls at each respective time point. The tests were one-way ANOVA with Dunnett’s post-test for normal distributed data or with Kruskal-Wallis with Dunn’s post-test for data that were not normal distributed (*p <0.05, **p<0.01 and ***p<0.001). The identities of all phosphatidylcholines were verified by the presence of the phosphatidylcholine fragment 184.07 m/z in the MS/MS analysis.

**Fig 8:**
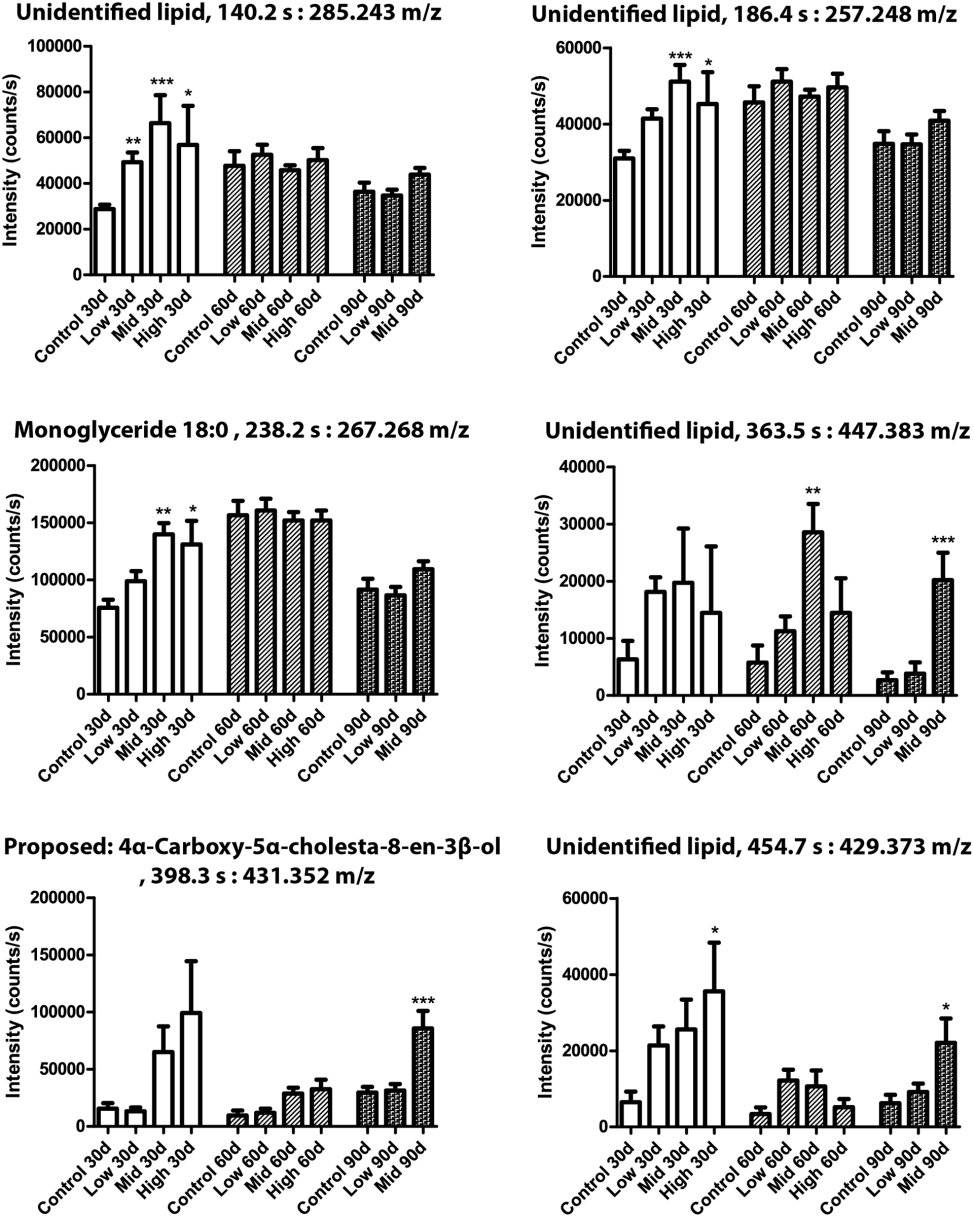
Neutral lipid metabolites were altered by the mixture. Metabolome analysis of rat blood after 30, 60 or 90 days of exposure. N = 10 for each group, Data are presented as mean ± SEM. The data were statistically tested as groups versus controls at each respective time point. The tests were one-way ANOVA with Dunnett’s post-test for normal distributed data or with Kruskal-Wallis with Dunn’s post-test for data that were not normal distributed (* designates p <0.05, **p<0.01 and ***p<0.001).

**Fig 9:**
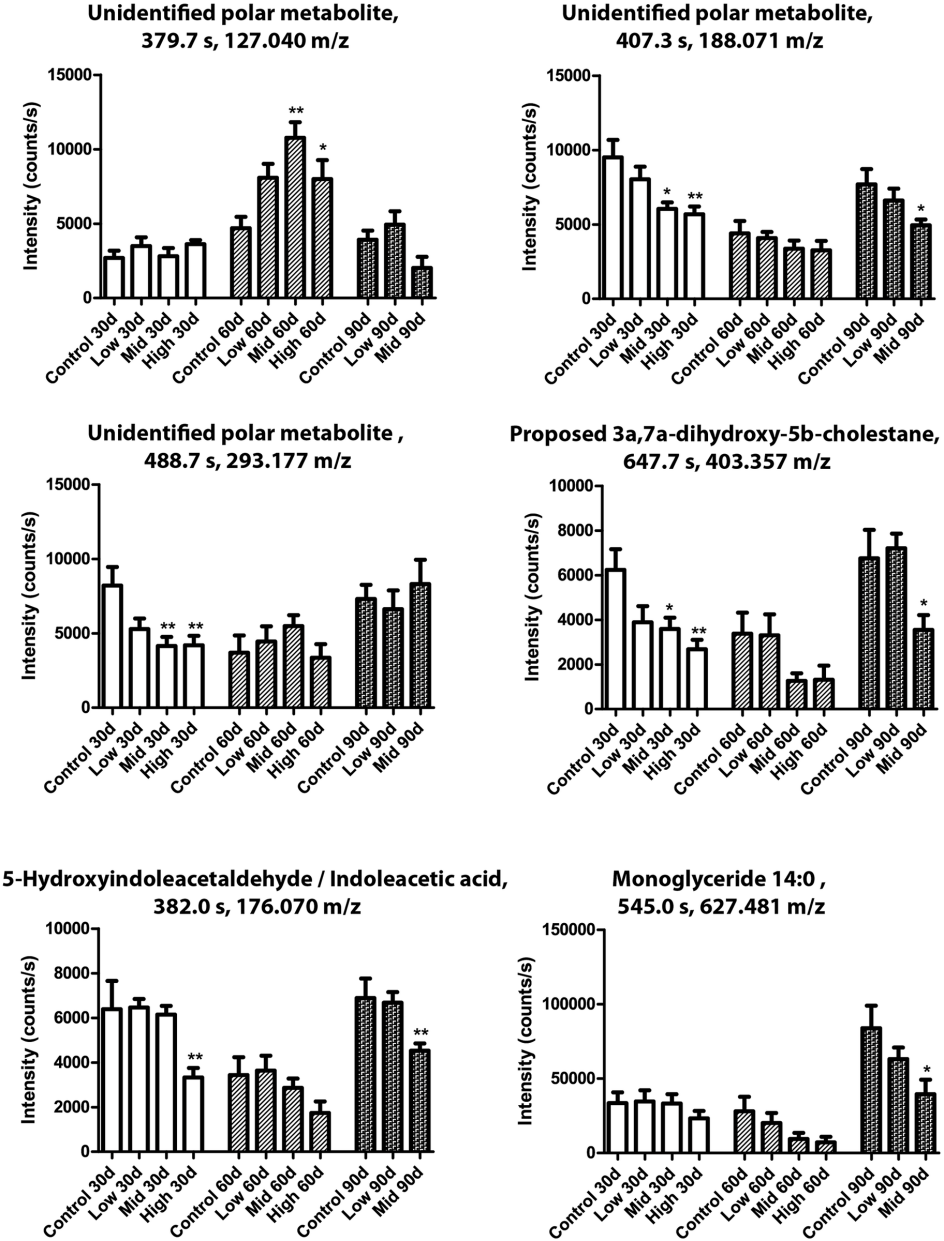
Polar metabolites were altered by the mixture. Metabolome analysis of rat blood after 30, 60 or 90 days of exposure. N = 10 for each group, Data are presented as Mean ± SEM. The data were statistically tested as groups versus controls at each respective time point. The tests were one-way ANOVA with Dunnett’s post-test for normal distributed data or with Kruskal-Wallis with Dunn’s post-test for data that were not normal distributed (*p <0.05, **p<0.01 and ***p<0.001).

Seven phosphatidylcholines were significantly regulated between various test groups and/or exposure duration (Fig 7). Phosphatidylcholine (18:0) was increased at all time points in Mid-dose, but decreased at 30 days and increased at 60 days with the High-dose. A similar expression pattern was observed for phosphatidylcholine (20:4) and phosphatidylcholine (22:6). Phosphatidylcholine (14:0, 16:0, 16:1) and phosphatidylcholine (18:2) were all decreased in High-dose animals at 30 days. Similarly, six metabolites from the neutral lipid fraction were found to be significantly changed between groups and exposure periods (Fig 8). For the neutral lipids no decreases were observed, but several metabolites were increased in response to chemical exposure. Practically all of the neutral lipids showed elevated levels after 30 days, albeit not all statistically significant, and all but the proposed metabolite 4α-Carboxy-5α-cholesta-8-en-3β-ol also in the Low-dose group. After 60 and 90 days the effects were less severe, and for the unidentified lipids at 285.243 and 257.248 m/z, as well as monoglyceride 18:0, any effects were negligible. The unidentified lipid at 447.383 m/z showed strongest increase in blood plasma from the Mid-dose animals. The monoglyceride 18:0 fragment was verified by identifying its acyl group fragment by MS/MS. Finally, metabolites from the polar fractions were typically decreased relative to control (Fig 9). One exception was metabolite (127.040 m/z), which was significantly increased after 60 days of exposure. Monoglyceride 14:0 was verified by the presence of Na^+^, H_2_O and acyl chain fragments, and was found to be decreased by Mid-dose at 90 days.

## Discussion

In this study, we aimed to test if a low-dose mixture containing 27 environmental chemicals left an adverse pathophysiological footprint in juvenile male rats. Exposing juvenile male rats for 3 months to a mixture of 27 chemicals resulted in liver injury. A dose-dependent increase in liver weights and histological changes were evident, indicative of hepatic steatosis which was further verified by a liver toxicity gene array. Changes to the metabolome were also detected in animals from the Low-dose group, potentially associated with hepatotoxicity.

Initially we analyzed the animals for various endpoints that we thought could be affected based on previous knowledge of individual chemicals. For instance, the chemical mixture included several reported endocrine disrupting chemicals such as PFNA [1], BPA [23] and phthalates [24]. Although endocrine disruptors often are most detrimental to male reproductive parameters during *in utero* development, we predicted some disrupting effects to hormone levels or testis maturation. Thus we measured testicular weight and selected steroid hormones. Surprisingly, we did not detect any significant changes in the serum levels of testosterone, androstenedione, corticosterone or progesterone. There was a trend towards elevated testosterone, but the data were not sufficiently robust to conclude on any real effects. Testis weights were also unchanged in the exposed animals and we conclude that the chemical mixture did not adversely affect these endpoints following three months of chronic exposure. Even though single chemicals included in the mixture are known to affect these endpoints, we observed no mixture effects at the selected doses and life-stage.

The three organs that showed increased weight in exposed animals were the spleen, kidney and liver. They were all affected in a dose-dependent manner and significantly larger in Mid-dose group compared to controls, with the liver also larger in the Low-dose group. Although kidneys and spleens displayed slight hypertrophy at higher exposure levels, we decided to look more closely at the livers only, as the overall aim of the study was to detect adverse effects by low-dose chemical exposure. The livers showed clear signs of steatosis, evident by excessive lipid accumulation in hepatocytes, with dose-dependent increase in severity and significant changes observed already at the lowest dose.

Signs of general hepatotoxicity were also evident at the transcriptional level. Several marker genes were dysregulated, including *Nqo1*, *Cyp1a2* and *Fmo1*. *Nqo1* encodes an antioxidant protection enzyme that is often up-regulated in livers of rodents exposed to hepatotoxicity-inducing chemicals and a strong indicator of liver injury [25][26]. So too, *Cyp1a2*, an Aryl hydrocarbon receptor (AHR)-responsive hepatic CYP gene, is a well-established liver marker for adaptive metabolic response to xenobiotics, likely serving to protect against hepatocellular damage and inflammation [27]. The flavin-containing monooxygenase encoding gene *Fmo1*, however, was down-regulated in the Mid-dose group. FMOs are known to metabolize a broad spectrum of foreign chemicals [28]. Thus the down-regulation in this study was surprising, but may indicate a compensatory mechanism reflecting increased enzymatic activities. Interestingly, loss of *Fmo1* in mice was recently shown to result in a lean phenotype in mice, thus being involved in the regulation of energy homeostasis [29]. We also observed a lower body weight in exposed animals. But whether there is a causal link between down-regulation of *Fmo1* and loss of body weight in our study cannot be determined.

We also performed an extensive metabolomics analysis of plasma collected at various times during the cause of the experiment. Metabolomics PLS plots suggested that the metabolome was changed already in the Low-dose group, with a more marked affect in the Mid-dose group. Effects were evident for phospholipids, neutral lipids and the polar fraction (for the positive ionization mode). Such changes in lipid homeostasis can be associated with liver toxicity, as the liver is a central organ in the synthesis and metabolism of these lipids. That is, the changes to the metabolome may simply reflect altered liver function [30]. In fact, altered lipid metabolism is a common response to chemical exposure [31][32] and it has been demonstrated that a stress-response to chemicals can lead to an increase in carbohydrate metabolism, subsequently causing alterations in lipid metabolism [32].

We observed general changes to the levels of phosphatidylcholines, with longer-chained phosphatidylcholines (phosphatidylcholine:18/20/22) typically increased, albeit a general decrease was observed at High-dose after 30 days of exposure for shorter chained phosphatidylcholines (phosphatidylcholine:14/16). Notably, this decrease at 30 days appears to be corrected already at 60 days of exposure for most phosphatidylcholines, suggesting that the body is capable of compensating for this early effect. The phosphatidylcholines are building blocks for cell membranes and participate in signal transduction [33]. Thus the observed changes in phosphatidylcholines indicate that these parameters were affected in the rats.

The neutral lipid fraction was also disturbed as illustrated by changes in proposed metabolites such as monoglyceride 14:0 and the acyl fragment of monoglyceride 18:0. Monoglycerides have been shown to participate in signalling transduction [34] but changes in these could also reflect general disturbance to lipid metabolism and thus be linked to the histologically observed steatosis. The proposed neutral lipid metabolite, 4α-Carboxy-5α-cholesta-8-en-3β-ol was found to be increased, indicating a disturbance in cholesterol biosynthesis [22]. A decrease of the proposed metabolite 3α,7α-dihydroxy-5β-cholestane in the polar fraction indicates that there is a dysregulation of bile acid synthesis [35].

It is notable that the prevalence of hepatic steatosis in the United States is now 20–40% [36,37]. This is a high incidence rate that could be partially attributed to an ever-increasing chemical burden [38]. In this study, we have shown clear signs of liver toxicity at the lowest exposure dose of the 27-chemical mixture; effects we deem unlikely to be the result of a single compound. It has been suggested that the Pareto’s rule, which states that 20% of the causal factors determine 80% of the effects, may apply also when determining the drivers of a mixture effect [39]. Regarding the constituents of our chemical mixture in relation to liver toxicity, four compounds could be flagged as such potential contributors to the liver toxicity: arsenic, bisphenol A, PCB and TCDD.

We observed signs of hepatotoxicity already in the Low-dose group, and we suggest that the accumulated dose of these four chemicals may be the drivers of the hepatotoxicity observed following prolonged exposure. Arsenic has been shown to increase liver peroxidation and absolute organ weight following exposure to 0.02 mg/kg bw/day for 60 days [40]. In our exposure regimen of 30 days, the arsenic dose in the Low-dose group was 20-fold lower, thus unlikely to have caused the observed liver toxicity on its own. Exposure to bisphenol A at 5 μg/kg bw for 10 weeks induced fat deposition in rat liver [41], whereas perinatal exposure to 50 μg/kg bw/day induced increased apoptosis in rat livers [42]. Our Low-dose of 10 μg/kg bw/day can thus be speculated to contribute to the observed liver toxicity. PCB153 has been shown to cause liver injury in mice following exposure to 195 μg/kg bw/day for 28 days, which gives an accumulated dose of 5.4 mg/kg bw [43]. In comparison, our accumulated Low-dose was 1.8 mg/kg bw (20 μg/kg bw/day). Moreover, in a subchronic rat toxicity study, PCB153 induced histological liver changes with a NOAEL of 34 μg/kg bw/day [44]. Thus, it is unlikely that our PCB153 dose induced the observed liver effects on its own. Finally, TCDD, has been reported to cause liver toxicity following exposure to 100 ng/kg bw/day for 13 weeks, resulting in an accumulated dose of 6.5 μg/kg bw [45]. Our accumulated dose following 3 month of exposure was estimated at 0.66 μg/kg bw (when taking the half-life and non-first order kinetics into account [46]). In another study [45], a dose of 10 ng/kg bw/day reportedly did not result in any adverse liver effects after 3 months of exposure, whereas a dose of 100 ng/kg bw/day did. Our Low-dose falls between these two doses, and therefore it cannot be excluded that TCDD in the present study contributes to the observed liver toxicity (or other adverse effects) in juvenile male rats. Taken together we deem it likely that it was the combined insult of these 4 contaminants in the entire mixture that adversely affected the liver.

We found that a low-dose mixture of 27 chemicals at a combined dose of 0.16 mg/kg bw/day left an adverse footprint in juvenile male rats. The mixture was designed to represent a combined dose approaching human exposure levels and we propose that our results show that the combined environmental load of a large number of chemicals can pose a risk to the general population. A few previous studies also support this notion. For instance, PFNA exposure as low as 12.5 μg/kg bw/day for 14 days induces adverse effects, both alone and when co-administered with a mixture of 14 environmental chemicals [1]. Others have found low-dose effects from mixtures on natural killer cell lytic activity [2], prostate inflammation in rats following exposure to an atrazine metabolite mixture at a dose of 0.09 mg/kg bw/day [3], and blood cell count changes in mice after exposure to a mixture of pesticides at doses derived from the acceptable daily intake levels in humans [4]. Changes to the blood cell count, as well as hemoglobin concentrations in mice following exposure to a mixture of 4, 5 and 8 μg/kg bw/day of atrazine, endosulfan and chlorpyrifos, respectively, has been reported [5], which support the observations from Merhi and co-workers [4]. Finally, additive effects of thyroid disrupters at a dose based on the environmental background has been reported in rats [6]. Hence, adverse effects can be induced when approaching human relevant levels of exposure. An important question to consider, however, is whether the chemical doses used in the present study were appropriate to obtain plasma levels comparable to human exposure.

To overcome the immense challenge of designing a chemical mixture study representative of real-life exposure to hundreds, if not thousands, of compounds simultaneously, we opted to use a total of 27 compounds, where some congeners were dosed to represent the combined burden of a chemical group, for instance BPA as a proxy for bisphenols in general. This approach has some inherent problems though, including the fact that individual chemicals, despite belonging to the same class of compounds, not necessarily have the same biomolecular or cellular effect. Also, doses were determined using linear extrapolation of doses from high-dose animal studies. The urine and plasma concentrations are not necessarily linearly affected across the used dose-ranges, thus overall effect data must be viewed in light of these uncertainties

Another important consideration is that our calculations were based on administration of single chemicals to animals. Thus, potential chemical toxicokinetic interactions causing individual ADMEs to change during prolonged exposure are not accounted for, and could lead to higher or lower plasma concentrations than expected. To analyze the minimum amount of chemicals the rats were exposed to during the main study, a simple excretion experiment was performed. After a single dose of Low- and Mid-dose mixture, the concentration of a subset of chemicals was analyzed in 24 hour urine. At Low-dose, the urinary concentrations from two individual rats were 0.3/0.49 ng/mL (μg/L) for triclosan and 5.0/11.2 ng/mL for benzophenone-3. These are lower than the human urine levels reported in NHANES, which are 13 and 22.9 μg/L for triclosan and benzophenone-3, respectively. For mono-n-butyl phthalate, we measured urine levels of 314/617 ng/mL, which are higher than the human urinary level of 24.6 μg/L urine reported in NHANES 2009. These data indicate that our initial calculations were reasonable, but failed to exactly predict internal dose levels for all the chemicals. However, the dose recovery in urine for triclosan (~1.5%), benzophenone-3 (~8.5%) and mono-n-butyl phthalate (~19%) were quite low and indicate that the majority was accumulated in the rats, excreted in feces or as other metabolites in urine. When calculating the excreted dose per kg body weight, the rats excreted 0.08, 0.22 and 11.8 μg/kg bw/day of triclosan, benzophenone-3 and mono-n-butyl phthalate, respectively after a single Low-dose and 0.23, 0.66 and 35 μg/kg bw/day of triclosan, benzophenone-3 and mono-n-butyl phthalate, respectively after a single Mid-dose. Thus, the rats excreted the three chemicals at similar levels as children and adolescents in the general Danish population [17,47], where the daily mean excretion were measured at 0.030, 0.034 and 2.5 μg/kg bw/day, and the observed maximum levels were about 1000, 100 and 10 times higher for triclosan, benzophenone-3 and mono-n-butyl phthalate, respectively. Taken together these aspects of metabolism and dose extrapolations caused us to cautiously denote our mixture as “low-dose”, but still based on human exposure levels.

Of final note, we did not apply risk assessment-based safety factors that are commonly done in animal studies. Such factors are typically in the order of 100-fold to account for animal-to-human extrapolation, as well as inter-individual differences. We also note that humans are likely exposed to thousands of chemicals simultaneously, not all being covered by the 27 chemicals included in this study. Based on this we suggest that the current findings raise concerns that the current chemical burden can pose a risk to the general population. We suggest increased emphasis on safety margins to protect humans against environmental chemicals.

## Supporting Information

S1 Table Chemical mixtures. List of chemical mixtures used in the exposure experiments, including all the constituent compounds and methods for dose calculations.
(DOC)

S2 Table Metabolite identification. Potential metabolites identified by comparing m/z ratios of metabolites with m/z ratios obtained from the human metabolome (HMDB) database. Abbreviations, Retention time (Ret. Time).
(DOC)
