# Supplementary material for: Juvenile Male Rats Exposed to a Low-Dose Mixture of Twenty-Seven Environmental Chemicals Display Adverse Health Effects
Source: PLoS One. 2016 Sep 6;11(9):e0162027. doi: 10.1371/journal.pone.0162027 (PMC5012700; doi:10.1371/journal.pone.0162027)
Supplement: S1 Table — List of chemical mixtures used in the exposure experiments, including all the constituent compounds and methods for dose calculations. (DOC) [file pone.0162027.s001.doc]

**S1 Table. Chemicals included in the mixture, with calculations of doses.**

| **Chemical**  **(common name /chemical name)** | **CAS No.** | **Concentration measured in human samples** | **Bioavailability study used to determine a dose for rats that in theory gives a plasma concentration similar to that observed in humans^&^** | **Low-dose group**  **(µg/kg bw/day)** |
| --- | --- | --- | --- | --- |
| Acrylamide | 79-06-1 | 5.4 nmol/L plasma ^#^ | A single dose of 100 µg/kg bw gave a serum conc. of 500 nmol/L in rats [1], thus a dose of 1 µg/kg bw/day could be suggested. A dose of 4 µg/kg bw/day was selected to also cover for the acrylamide metabolite glycidamide in humans. | 4 |
| Benzophenone-3 /oxybenzone | 131-57-7 | 22.9 µg/L urine ^#^ | A dose of 100 mg/kg bw (**value A**)^&^ given to rats gave a urine conc. of 0.9 mg/mL [2] (**value B**)^&^. When considering a value of 22.9 µg/L urine in humans, a 39,000-fold lower dose than what used by Kadry *et al.* [2] can be proposed (0.9 mg/mL / 22.9 µg/L). Thus a suggested dose for the present study was: 100 mg/kg bw / 39,100 x = 0.0026 mg/kg bw/day. | 2.6 |
| Bisphenol A | 80-05-7 | 2.64 µg/L urine ^#^. Alternatively a plasma concentration in the Hong Kong population was 0.95 µg/L [3]. | A^&^: 10 mg/kg bw in rats; B^&^: 30 ng/mL plasma [4].  = 0.31 mg/kg bw/day  This was considered a larger than expected dose. The human exposure reported by EFSA is 0.04-1.5 µg BPA/kg bw/day, through conversion by body surface area yielding a dose of 10 µg/kg bw/day in rats. This dose was selected to also cover exposure to other bisphenols. | 10 |
| Triclosan | 3380-34-5 | 13 µg/L urine  ^#^ | A^&^: 4 mg to humans; B^&^: 13.3 µg/L urine [5].  = 4 mg/kg bw/day, corresponding to 0.07 mg/kg bw/day in a 60 kg person and – corrected for body surface area (conversion factor 6.2) [6] corresponds to a dose of 0.41 mg/kg bw/day in rats.  However, human intake has been estimated to be 0.2 µg/kg bw/day in geometric mean and 0.8 in 95th percentile [7]. This gives a rat dose (conversion factor body surface area x6.2) of 1.2 and 5 µg/kg bw/day. | 5 |
| Ortho-phenylphenol | 90-43-7 | 0.5 µg/L urine  ^#^ | A^&^: 933 mg/kg bw to rats; B^&^: 7480 mg/L urine [8].  = 0.062 µg/kg bw/day | 0.06 |
| trans-nonachlor | 39765-80-5 | 0.11 ng/g serum  ^#^ | Trans–nonachlor is a metabolite of chlordane. A^&^: Chlordane at 100 µg/kg bw to rats ; B^&^: chlordane of 10 ng/mL serum [9].  = 1.1 µg/kg bw/day. This dose was erroneously decreased another 3-fold. | 0.35 |
| p.p-DDE / Dichlorodiphenyldichloroethylene | 72-55-9 | 1.54 ng/g serum ^#^ | A^&^: 5 mg DDE /kg bw/day to rats; B^&^: 561 ng/mL plasma [10]. | 13 |
| 2.4.6-trichlorophenol | 88-06-2 | 2.85 µg/L urine ^#^ | Lindane was used as a measure of 2.4.6-trichlorophenol.A^&^: 68 mg/kg bw/day to rats; B^&^: 8 mg/L urine [11].  = 0.024 mg/kg bw/day.  Due to differences in lipophilicity between lindane and trichlorphenol a dose of 10 µg/kg bw/day was selected | 10 |
| Chlorpyrifos | 2921-88-2 | 1.77 µg/L urine of the metabolite TCPγ.  ^#^ | A^&^: 5 mg/kg bw of chlorpyrifos to rats B^&^: TCPγ at 23.76 mg/L urine [12]. | 0.4 |
| 3-phenoxybenzoic acid | 3739-38-6 | 0.29 µg/L urine ^#^ | A^&^: 0.76 mg/kg bw to rats; B^&^: 20 mg/L urine [13]. | 0.01 |
| Arsenic | 1327-53-3 | 8.3 µg/L urine  ^#^ | A^&^: 5 mg/kg bw/day to rats ; B^&^: 35 mg/L urine [14].  = 1.2 µg/kg bw/day. This arsenic dose was given as arsenic trioxide at a dose of 3.1 µg/kg/day. | 1.2 |
| Barium | 10361-37-2 | 1.5 µg/L urine  ^#^ | A^&^: 4 µg / kg bw/day to rats; B^&^: 45 µg/L urine [15].  = 0.13 µg/kg/day. This barium dose was given as bariumchloride, 2H_2_O at a dose of 0.23 µg/kg/day. | 0.13 |
| Cadmium | 10108-64-2 | 0.41 µg/L blood  ^#^ | A^&^: 0.1 mg/kg bw to rat pups; B^&^: 400 µg/L blood [16].  = 0.1 µg/kg/day. This cadmium dose was given as cadmiumchloride at a dose of 0.16 µg/kg/day | 0.1 |
| Cesium | 7647-17-8 | 4.4 µg/L urine ^#^ | A^&^: 1 mg/kg bw/day to rats; B^&^: 9 mg/L Urine [17].  = 0.48 µg/kg/day.  This dose was given as cesiumchloride at a dose of 0.61 µg/kg/day. | 0.48 |
| Cobalt | 7646-79-9 | 0.38 µg/L urine  ^#^ | A^&^: 1 mg/kg bw/day to rats; B^&^: 7 mg/L urine [18].  = 0.05 µg/kg bw/day µg/kg.  This cobalt dose was given as cobalt(11)chloride at dose of 0.11 µg/kg/day. | 0.05 |
| Lead | 7758-95-4 | 0.80 µg/L urine  ^#^ | A^&^: 20 mg/kg bw/day to rats ; B^&^: 1 mg/L urine [19].  = 16 µg/kg bw/day.  This lead dose was given as leadchloride at a dose of 21.5 µg/kg/day. | 16 |
| Mercury | 7487-94-7 | 0.44 µg/L urine  ^#^ | A^&^: 1 mg/kg bw/day to rats; B^&^: 125 µg/L urine [20].  = 3.5 µg/kg bw/day.  This mercury dose was given as mercury(11)chloride at a dose of 4.7 µg/kg/day. | 3.5 |
| Thallium | 7791-12-0 | 0.18 µg/L urine  ^#^ | A^&^: 8 mg/kg bw to rats; B^&^: 5.1 mg/L urine [21].  = 0.3 µg/kg bw/day.  This thallium dose was given as thallium(1)chloride at a dose of 0.35 µg/kg/day. | 0.3 |
| PFOS / Perfluoro-octanesulfonic acid | 111873-33-7 | 20.7 µg/L serum  ^#^ | A PFNA dose of 0.0125 mg/kg bw/day gave a plasma concentration of 400 µg/L in rats [22]. The T½’s of PFOS and PFNA in male rats are 25 and 31 days, respectively, thus within the same range. This gives a proposed dose of 0.6 µg/kg bw/day, but to cover other congeners a dose of 0.9 µg/kg bw/day was selected. | 0.9 |
| PFNA / Perfluoro-nonanoic acid | 375-95-1 | 1.0 µg/L serum ^#^ | A PFNA dose of 0.0125 mg/kg bw/day gave a plasma concentration of 400 µg/L [22]. That gives a proposed dose of 0.03 µg/kg bw/day, but to cover other congeners a dose of 0.2 µg/kg bw/day was selected. | 0.2 |
| Mono-n-butyl phthalate | 131-70-4 | 24.6 µg/L urine ^#^ | To cover all phthalates the COT Statement on dietary exposure to phthalates [23] was used.  Here the 97.5th percentile of the external exposure estimates in µg/kg bw/day for 6/7 year old children was used. A total exposure of DEHP (6.7), DBP (0.7), DIBP (1.8), BBP (0.9) was 10 µg/kg bw/day and using a body surface correction factor of 6.2 [6] this provides a dose of 62 µg/kg bw/day to rats | 62 |
| AHTN / 6-Acetyl-1,1,2,4,4,7-hexamethyltetraline | 1506-02-1 | No data on human tissue concentrations. Values are based on intake data [24]. | According to the HERA Risk Assessment of AHTN [24], a daily intake through fish consumption is 1 μg/kg bw/day in humans. Calculating via body surface area [6]. body surface area conversion article) into a rat dose then we get a dose of 6.2 µg/kg bw/day | 6.2 |
| PCB 153 (covering the PCBs) /  Polychlorinated biphenyl 153 | 35065-27-1 | 0.17 ng/g serum ^#^  An alternative number is reported by Bakker *et al.*[25]. Here seven indicator PCBs are present at 0.36 ng/g serum | A^&^: arochlor (a commercial PCB product) at 25 mg/kg bw to rats; B^&^: gave a concentration of two metabolites of a total of 180 ng/g plasma [26]. | 20 |
| TCDD (Dioxines) / 2,3,7,8-Tetrachlorodibenzo-p-dioxin | 1746-01-6 | 2.6 ng/L serum (sum of four dioxins: HpCCD 155 fg/g serum; HxCCD 105 fg/g serum; OCDD 2230 fg/g serum; HpCDF 62 fg/g serum) ^#^ | A^&^: 0.05 µg/kg bw to rats; B^&^: 1.2 ng/L plasma [27].  = 0.1 mg/kg bw/day.  The dose was erroneous calculated to be 2.2 fold *lower* rather than 2.2 fold *higher* and the used dose was 0.034 µg/kg bw/day. | 0.034 |
| Benzo[a]pyrene (PAHs) | 50-32-8 | 6.3 µg/L urine  (a sum of 10 PAHs: 2-hydroxy-fluorene 304 ng/L; 3-hydroxy-fluorene 134 ng/L; 9-hydroxy-fluorene 267 ng/L; 1-hydroxy-naphtalene 2680 ng/L; 2- hydroxyl-naphtalene 2470 ng/L; 1-hydroxy-phenantrene 140 ng/L; 2-hydroxy-phenantrene 54 ng/L; 3-hydroxy-phenantrene 105 ng/L; 4-hydroxy-phenantrene 23 ng/L; 1-hydroxy-pyrene 89 ng/L) ^#^ | A^&^: 100 mg/kg bw to rats; B^&^: 750 mg/L urine [28].  = 0.8 µg/kg bw/day.  However, when taking into account that benzo[a]pyrene is the most toxic congener of the PAHs a dose of 0.4 µg/kg bw/day was proposed. | 0.4 |
| PHIP / 2-Amino-1-methyl-6-phenylimidazo(4,5-b)pyridine | 105650-23-5 | 0.41 ng/ L (mean of 13 subjects, 24 h urine set to 1 L) based on [29] | A^&^: 40 mg/kg bw to rats; B^&^: 10 mg/L urine [30].  = 0.0016 µg/kg bw/day.  In addition a calculation via food intake in humans was conducted. According to Nagao *et al.* [31] it is approximately 0.09 µg/kg bw and conversion by body surface area from humans to rats (x6.2) this gives a value of 0.5 µg/kg bw/day.  Taking both calculations into account a dose of 0.1 µg/kg bw/day was selected. | 0.1 |
| MeIQx /  2-Amino-3,8-dimethylimidazo[4,5-f]quinoxaline | 77500-04-0 | 22.5 ng/ L (mean of 13 subjects, 24 h urine set to 1 L) based on [29] | A^&^: 18.8 mg/kg bw to rats; B^&^: 26 mg/L urine [30].  = 0.016 µg/kg bw/day.  In addition a calculation via food intake in humans was conducted. According to Nagao *et al.* [31] it is approximately 0.02 µg/kg bw and conversion by body surface area from humans to rats (x6.2) which gives a value of 0.1 µg/kg bw/day.  Taking both calculations into account a dose of 0.05 µg/kg bw/day was selected. | 0.05 |
| **Total dose** |  |  |  | **160** |

^#^ Geometric mean [32]. ^&^ The calculations for Benzophenone-3 serves as an example. For most other chemicals a similar calculation was conducted. Only the reference used with the dose given to a given species (value A) and the urine/plasma concentration (value B) are given for these chemicals

References

1. Berger FI, Feld J, Bertow D, Eisenbrand G, Fricker G, Gerhardt N, et al. Biological effects of acrylamide after daily ingestion of various foods in comparison to water: A study in rats. Mol Nutr Food Res. 2011;55: 387–399. doi:10.1002/mnfr.201000234

2. Kadry AM, Okereke CS, Abdel-Rahman MS, Friedman MA, Davis RA. Pharmacokinetics of benzophenone-3 after oral exposure in male rats. J Appl Toxicol. 1995;15: 97–102. doi:10.1002/jat.2550150207

3. Wan HT, Leung PY, Zhao YG, Wei X, Wong MH, Wong CKC. Blood plasma concentrations of endocrine disrupting chemicals in Hong Kong populations. J Hazard Mater. 2013;261: 763–769. doi:10.1016/j.jhazmat.2013.01.034

4. Upmeier A, Degen H, Diel P, Michna H. Toxicokinetics of bisphenol A in female DA / Han rats after a single i . v . and oral administration. Arch Toxicol. 2000;74: 1996–2001. doi:10.1007/s002040000144

5. Sandborgh-Englund G, Adolfsson-Erici M, Odham G, Ekstrand J. Pharmacokinetics of triclosan following oral ingestion in humans. Journal of toxicology and environmental health. Part A. 2006. doi:10.1080/15287390600631706

6. Reagan-Shaw S, Nihal M, Ahmad N. Dose translation from animal to human studies revisited. FASEB J. 2007/10/19 ed. 2008;22: 659–661. doi:10.1096/fj.07-9574LSF

7. Rodricks J V, Swenberg JA, Borzelleca JF, Maronpot RR, Shipp AM. Triclosan: a critical review of the experimental data and development of margins of safety for consumer products. Crit Rev Toxicol. 2010;40: 422–484. doi:10.3109/10408441003667514

8. Nakao T, Ushiyama K, Kabashima J, Nagai F, Nakagawa A, Ohno T, et al. The metabolic profile of sodium o-phenylphenate after subchronic oral administration to rats. Food Chem Toxicol. 1983;21: 325–329. doi:10.1016/0278-6915(83)90068-6

9. Ohno Y, Kawanishi T, Takahashi A, Nakaura S, Kawashima K, Tanaka S, et al. Comparisons of the toxicokinetic parameters in rats determined for low and high dose of gamma-chlordane. J Toxicol Sci. 1986;11: 111–123. doi:10.2131/jts.11.111

10. Leavens TL, Sparrow BR, Devito MJ. Lack of antiandrogenic effects in adult male rats following acute exposure to 2,2-bis(4-chlorophenyl)-1,1-dichloroethylene (p,p???-DDE). Toxicology. 2002;174: 69–78. doi:10.1016/S0300-483X(02)00072-0

11. Baliková M, Kohlicek J, Rybka K. Chlorinated phenols as metabolites of lindane. Evaluation of the degree of conjugation in rat urine. J Anal Toxicol. 13: 27–30.

12. Smith JN, Campbell JA, Busby-Hjerpe AL, Lee S, Poet TS, Barr DB, et al. Comparative chlorpyrifos pharmacokinetics via multiple routes of exposure and vehicles of administration in the adult rat. Toxicology. 2009;261: 47–58. doi:10.1016/j.tox.2009.04.041

13. Crayford J V, Hutson DH. The metabolism of 3-phenoxybenzoic acid and its glucoside conjugate in rats. Xenobiotica. 1980;10: 355–364. doi:10.3109/00498258009033768

14. Naranmandura H, Suzuki N, Iwata K, Hirano S, Suzuki KT. Arsenic metabolism and thioarsenicals in hamsters and rats. Chem Res Toxicol. 2007;20: 616–624. doi:10.1021/tx700038x

15. Gonzalez-Reimers E, Rodriguez-Moreno F, Martinez-Riera A, Mas-Pascual A, Delgado-Ureta E, Galindo-Martin L, et al. Relative and combined effects of ethanol and protein deficiency on strontium and barium bone content and fecal and urinary excretion. Biological trace element research. 1999. doi:10.1007/BF02784395

16. Eklund G, Petersson Grawé K, Oskarsson A. Bioavailability of cadmium from infant diets in newborn rats. Arch Toxicol. 2001;75: 522–530. doi:10.1007/s00204-001-0280-z

17. HOOD SL, COMAR CL. Metabolism of cesium-137 in rats and farm animals. Arch Biochem Biophys. 1953;45: 423–433. doi:10.1016/S0003-9861(53)80018-4

18. Firriolo JM, Ayala-Fierro F, Sipes IG, Carter DE. Absorption and disposition of cobalt naphthenate in rats after a single oral dose. J Toxicol Environ Health A. 1999;58: 383–395. doi:10.1080/009841099157223

19. Victery W, Miller CR, Zhu SY, Goyer RA. Effect of different levels and periods of lead exposure on tissue levels and excretion of lead, zinc, and calcium in the rat. Toxicol Sci. 1987;8: 506–516. doi:10.1093/toxsci/8.4.506

20. Burk RF, Jordan HE, Kiker KW. Some effects of selenium status on inorganic mercury metabolism in the rat. Toxicol Appl Pharmacol. 1977;40: 71–82.

21. Pedro A, Lehmann F, Favari L. Acute thallium intoxication: kinetic study of the relative efficacy of several antidotal treatments in rats. Arch Toxicol. 1985;57: 56–60. doi:10.1007/BF00286576

22. Hadrup N, Pedersen M, Skov K, Hansen NL, Berthelsen LO, Kongsbak K, et al. Perfluorononanoic acid in combination with 14 chemicals exerts low-dose mixture effects in rats. Arch Toxicol. 2015/01/16 ed. 2015; doi:10.1007/s00204-015-1452-6

23. COT. COT STATEMENT ON DIETARY EXPOSURE TO PHTHALATES – DATA FROM THE TOTAL DIET STUDY (TDS). 2010. doi:http://cot.food.gov.uk/sites/default/files/cot/cotstatementphthalates201104.pdf

24. HERA. HERA Risk Assessment of AHTN (6-Acetyl-1,1,2,4,4,7-hexamethyltetraline). http://www.heraproject.com/files/28-hh-04-pcm ahtn hera human health discl ed2.pdf [Internet]. 2004. Available: http://www.heraproject.com/files/28-hh-04-pcm ahtn hera human health discl ed2.pdf

25. Bakker M, Baars A, Baumann R, Boon P, Hoogerbrugge R. Indicator PCBs in foodstuffs: occurrence and dietary intake in The Netherlands at the end of the 20th century. http://rivm.openrepository.com/rivm/bitstream/10029/9046/1/639102025.pdf [Internet]. 2003. Available: http://rivm.openrepository.com/rivm/bitstream/10029/9046/1/639102025.pdf

26. Bergman A, Klasson-Wehler E, Kuroki H. Selective retention of hydroxylated PCB metabolites in blood. Environ Health Perspect. 1994;102: 464–469. doi:10.1289/ehp.94102464

27. Hurst CH, DeVito MJ, Setzer RW, Birnbaum LS. Acute administration of 2,3,7,8-tetrachlorodibenzo-p-dioxin (TCDD) in pregnant Long Evans rats: association of measured tissue concentrations with developmental effects. Toxicol Sci. 2000;53: 411–420.

28. Ramesh A, Inyang F, Hood DB, Archibong AE, Knuckles ME, Nyanda AM. Metabolism, bioavailability, and toxicokinetics of benzo(alpha)pyrene in F-344 rats following oral administration. Exp Toxicol Pathol. 2001;53: 275–290.

29. Wakabayashi K, Ushiyama H, Takahashi M, Nukaya H, Kim SB, Hirose M, et al. Exposure to heterocyclic amines. Environmental Health Perspectives. 1993. pp. 129–134. doi:10.1289/ehp.9399129

30. Gerbl U, Cichna M, Zsivkovits M, Knasmüller S, Sontag G. Determination of heterocyclic aromatic amines in beef extract, cooked meat and rat urine by liquid chromatography with coulometric electrode array detection. J Chromatogr B Anal Technol Biomed Life Sci. 2004;802: 107–113. doi:10.1016/j.jchromb.2003.10.063

31. Nagao M, Wakabayashi K, Ushijima T, Toyota M, Totsuka Y, Sugimura T. Human exposure to carcinogenic heterocyclic amines and their mutational fingerprints in experimental animals. Environmental Health Perspectives. 1996. pp. 497–501. doi:10.1289/ehp.96104s3497

32. NHANES. Fourth National Report on Human Exposure to Environmental Chemicals. http://www.cdc.gov/exposurereport/ [Internet]. 2009. Available: http://www.cdc.gov/exposurereport/
