# Supplementary material for: Juvenile Male Rats Exposed to a Low-Dose Mixture of Twenty-Seven Environmental Chemicals Display Adverse Health Effects
Source: PLoS One. 2016 Sep 6;11(9):e0162027. doi: 10.1371/journal.pone.0162027 (PMC5012700; doi:10.1371/journal.pone.0162027)
Supplement: S2 Table — Potential metabolites identified by comparing m/z ratios of metabolites with m/z ratios obtained from the human metabolome (HMDB) database. Abbreviations, Retention time (Ret. Time). (DOC) [file pone.0162027.s002.doc]

**S2 Table. Metabolite identification.** Potential metabolites identified by comparing m/z ratios of metabolites with m/z ratios obtained from the human metabolome (HMDB) database. Abbreviations, Retention time (Ret. Time).

| **Metabolite m/z (Da)** | **HMDB m/z (Da)** | **m/z error (Da)** | **Ret. Time (s)** | **Potential identity_______________________________________________** |
| --- | --- | --- | --- | --- |
| *Phospholipid fraction* | | | | |
| 468.308 | 468.3085 | 0.0005 | 104.4 | Phospatidylcholine 14:0 [M+H] |
| 524.372 | 524.3711 | 0.0009 | 219.3 | Phospatidylcholine 18:0 [M+H] |
| 496.341 | 496.3398 | 0.0012 | 148.5 | Phospatidylcholine 16:0 [M+H] |
| 494.324 | 494.3241 | 0.0001 | 114.8 | Phospatidylcholine 16:1 [M+H] |
| 568.340 | 568.3398 | 0.0002 | 118.1 | Phospatidylcholine 22:6 [M+H] |
| 544.341 | 544.3398 | 0.0012 | 121.6 | Phospatidylcholine 20:4 [M+H] |
| 520.341 | 520.3398 | 0.0012 | 128.1 | Phospatidylcholine 18:2 [M+H] |
| *Neutral lipid fraction* | | | | |
| 285.243 | 285.2467 | 0.003704 | 140.2 | Unidentified [M+H] |
| 267.268 | n/a | n/a | 238.2 | Monoglyceride 18:0 (acyl chain fragment; adduct at +18.01) |
| 257.248 | 257.2475 | 0.0005 | 186.4 | Hexadecanoic acid / Trimethyltridecanoic acid [M+H] (adduct at +56.03) |
| 447.383 | 447.3833 | 0.0003 | 363.5 | 13'-Hydroxy-α-tocopherol / 13'-Carboxy-γ-tocopherol [M+H] (adduct at +56.03) |
| 431.352 | 431.3520 | 0.0000 | 398.3 | 4α-Carboxy-5α-cholesta-8-en-3β-ol [M+H] (MS/MS fragments: 167.07 and 207.1001 m/z) |
| 429.373 | 429.3727 | 0.0003 | 457.7 | 4α-Formyl-4β-methyl-5α-cholesta-8-en-3β-ol, 4α-Hydroxymethyl-4β-methyl-5α-cholesta-8,24-dien-3β-ol, and cholesteryl acetate, [M+H] |
| *Polar fraction* | | | | |
| 127.040 |  |  | 379.7 | Unidentified |
| 188.071 | 207.0895 | 0.0001 | 407.3 | 3-Indolepropionic acid/Indole-3-methyl acetate [M-H] |
| 403.357 | 403.3582 | 0.0012 | 647.7 | 3α,7α-Dihydroxy-5β-cholestane [M-H] |
| 293.177 | 293.1758 | 0.0012 | 488.7 | Unidentified |
| 627.481 | 627.4806 | 0.0004 | 545.0 | Monoglyceride 14:0 / [2M+Na] a fragment at -Na, -H2O and -acyl chain) |
| 176.070 | 176.0706 | 0.0006 | 382.0 | 5-Hydroxyindoleacetaldehyde / Indoleacetic acid [M+H] |
